# Design and Characterization of Sheet-Based Gyroid Porous Structures with Bioinspired Functional Gradients

**DOI:** 10.3390/ma13173844

**Published:** 2020-08-31

**Authors:** Yuan Jin, Haoyu Kong, Xueyong Zhou, Guangyong Li, Jianke Du

**Affiliations:** 1 School of Mechanical Engineering and Mechanics, Ningbo University, Ningbo 315211, China; konghaoyu@nbu.edu.cn (H.K.); zhouxueyong@nbu.edu.cn (X.Z.); liguangyong@nbu.edu.cn (G.L.); 2 State Key Laboratory of Fluid Power and Mechatronic Systems, School of Mechanical Engineering, Zhejiang University, Hangzhou 310027, China

**Keywords:** triply periodic minimal surfaces, branched sheet structures, functionally gradients, surface area to volume ratio, mechanical properties

## Abstract

A new type of sheet porous structures with functionally gradients based on triply periodic minimal surfaces (TPMS) is proposed for designing bone scaffolds. The graded structures were generated by constructing branched features with different number of sheets. The design of the structure was formulated mathematically and five types of porous structure with different structural features were used for investigation. The relative density (RD) and surface area to volume (SA/V) ratio of the samples were analyzed using a slice-based approach to confirm their relationships with design parameters. All samples were additively manufactured using selective laser melting (SLM), and their physical morphologies were observed and compared with the designed models. Compression tests were adopted to study the mechanical properties of the proposed structure from the obtained stress–strain curves. The results reveal that the proposed branched-sheet structures could enhance and diversify the physical and mechanical properties, indicating that it is a potential method to tune the biomechanical properties of porous scaffolds for bone tissue engineering (TE).

## 1. Introduction

In tissue engineering (TE), porous scaffolds are better candidates for the use of implants than solid materials due to their controllable biomechanical properties [1]. The high biocompatibility and non-allergic tissue response enable them to be widely used in the treatment of coronary defects [2]. One of the largest challenges in designing and manufacturing porous structures in the field of TE is to tailor their mechanical and biological properties for minimizing the stress shieling effect and possessing porosity morphology to allow tissue regeneration within the scaffold [3,4,5]. To address the mismatch in elastic properties between implants and surrounding tissues, the Young’s modulus of the scaffold should be optimized from both the material and structural sides [6,7]. Among them, cellular structures with interconnected and continuous pores fabricated by a wide range of materials have been developed for TE applications to achieve tunable mechanical properties, as well as desirable biological performance [8,9]. Clearly, the controllability over the geometric features such as pore size, porosity, pore shape and pore interconnectivity plays a significant role in designing cellular-based patient-specific multifunctional scaffolds [10].

The internal architecture design provides an effective strategy to control cellular-based scaffolds’ mechanical properties [11], thus hierarchical or multiscale porous scaffold with varying properties can be generated and fabricated thanks to the advent of additive manufacturing (AM) technology [12,13]. To fully mimic the intricate architecture and hierarchical property of human tissues, functionally graded cellular structures based on expected distributions of geometric features has been developed due to their outperformed abilities to tailoring biomechanical properties [14,15,16]. Functionally graded scaffolds (FGS) with variation in pore size, porosity, pore type and mechanical properties can not only be capable of mimicking the complex internal structure but also effectively achieve a balance between the biological efficiency and mechanical integrity [17,18]. With the use of computational modeling methods [19], FGS can be designed with controlled grading patterns using different unit cell types to combine all associated advantages into one scaffold structure [20].

Triply periodic minimal surfaces (TPMS) have received increasing attentions as they can be used for designing 3D interconnected and continuous porous structures with a mathematically-defined method [21]. The geometric features of TPMS, such as surface and topology are all mathematically defined, and thus can be tuned quantitatively based on the mathematical expressions. TPMS-based cellular scaffolds can be generated combining the volume distance field and Boolean operation [22], which enable to construct complex internal porous structures for arbitrary external shape of objects. Therefore, the porosity distribution associated with pore size and pore shape can be spatially and continuously varied based on the target functions from the design requirements. Moreover, the smooth surfaces and highly interconnected structures of TPMS-based scaffolds allow nutrients to transport towards the interior of the scaffold to facilitate cell ingrowth, vascularization, as well as waste material removal [23].

TPMS-based structures can be found in nature [21] and have been studied for decades [24]. It becomes an effective tool for designing porous scaffold in recent years for its easy controllability of internal pore architectures. The capability to achieve complex and intricate architectures via a layer-upon-layer paradigm of AM has enabled evolution of scaffold designs. The entire design process based on the target properties can be fully realized using the parametric design method without too many limitations from the manufacturing methods due to the high precision and reproducibility of AM. Grading structures where either porosity or pore shape varies throughout the scaffold structure can be readily generated by defining level parameters for changing the pore size or coefficients for tuning the cell size spatially [15]. Many researchers are devoted to creating hybrid [22,25,26], multi-scale [27], grading [6] and even stochastic [28] structures for applications in different fields based on TPMS.

The TPMS-based structures can be categorized into two types: network structure and sheet structure based on the generation process [29]. The space is partitioned into two sub spaces by a smooth and non-intersecting surface and the network structure can be formed when one of the generated sub space is filled with solid materials, while the other sub space is left empty. On the other hand, the sheet structure can be designed with two parallel TPMSs. When the intermediate space between them is filled with solid materials, two intertwined and interconnected pores would appear separated by the solid wall. Both structures have been utilized to design FGS using similar methodologies based on the implicit function of TPMS [30]. The greatest advantage of sheet-based structure over network-based structure is the higher surface area to volume ratio (SA/V ratio), which is a significant parameter that affects a variety of biological properties [31]. An increased SA/V ratio means more exposure to environment for more interaction with the surrounding environment. In some certain circumstances, the scaffold is supposed to provide sufficient surface for cell adhesion and regeneration of the host tissues [32], while a low SA/V ratio is expected in other circumstances to achieve better mechanical integrity. Hence, there remains a need for designing TPMS-based FGS with varying SA/V ratio.

In the present study, a new type of FGS is designed based on TPMS sheet structure with varying sheet numbers as a potential approach to satisfy some specific requirements in the field of bone TE, such as grading SA/V ratio and branched features. Compared with conventional porous scaffolds [33,34], TPMS-based scaffolds have remarkably high cell viability due to its unique and mathematically defined pore morphologies. The enhanced cell adhesion, migration and proliferation of TPMS-based scaffolds are directly related to its high SA/V ratio, thus the gradient on the sheet numbers contributes to the enhancement and tunability of these capabilities of designed bone scaffolds. Gyroid, one of the most popular TPMS, was utilized here due to its excellent mechanical integrity and porous morphology. Branched structures that are commonly seen in human body, such as coronary arterial tree [35] and pulmonary trachea [36], are essential for efficient vascular transport and tissue specific functions [37]. Inspired by this bionic structure, the TPMS-based scaffold was designed into a branched topology to achieve complex, multi-scale and grading porous structures. The modeling process of the FGS was provided based on the mathematical description of TPMS. Additionally, the designed samples were additively fabricated using selective laser melting (SLM), and then evaluated in terms of their structural characteristics and mechanical performance under compression. The results reveal that the proposed FGS could effectively generate branched sheet structures with tunable biomechanical properties and hence could be potentially adopted for designing bone scaffolds to meet increasing requirements on complexity and versatility.

## 2. Methodologies

### 2.1. Design of Sheet Structures Based on TPMS

**Figure 1:**
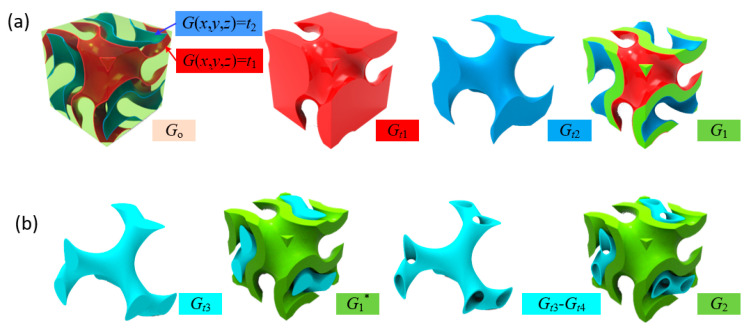
(**a**) Generation of TPMS-based sheet structure; and (**b**) generation of multi-sheet structure.

The Gyroid surface can be expressed as [38]:

(1) $$G=\sin\left(\frac{2\pi }{a}\times x\right)\cos\left(\frac{2\pi }{b}\times y\right)+\sin\left(\frac{2\pi }{b}\times y\right)\cos\left(\frac{2\pi }{c}\times xz\right)+\sin\left(\frac{2\pi }{c}\times z\right)\cos\left(\frac{2\pi }{a}\times x\right)=t$$

where constants *a*, *b* and *c* govern the unit cell size in three directions [39], and they are commonly the same in the designing process to obtain isotropic properties; the constant *t* controls the ratio of two volumes separated by the Gyroid surface and is termed as level parameter in this study. The sheet-based Gyroid structure can be generated from two individual and non-intersected surfaces with different level parameters via a Boolean difference operation between two volumes (*G_t_*_1_ and *G_t_*_2_) surrounded by the surfaces as illustrated in Figure 1. Thus, the intermediate zone between the two surfaces (*G*_1_) is filled with solid and can be described as follows:

(2) $$G_{1}=G_{o}{⋂}^{}\left(G_{t1}-G_{t2}\right)$$

where *G_o_* is the volume defined by the external shape of target object. The sheet thickness is determined by the two level parameters (*t*_1_ and *t*_2_) and can be defined as a function of location vector to achieve a graded porous structure [40]. Based on the generated sheet structure in Figure 1a, an extra network (*G_t_*_3_) or sheet (*G_t_*_3_–*G_t_*_4_) structure can fill inside without any intersection with the original sheet structure, as illustrated in Figure 1b. The extra structure results in the increase of the SA/V ratio to provide more surface for cell attachment and can be potentially adopted to construct grading geometric features.

The level parameters control the topology and wall thickness of TPMS-based sheet structures, so the multi-sheet structure can be described according to the level parameters. A two-sheet structure can be described as follows:

(3) $$G_{2}=G_{o}{⋂}^{}(\left(G_{t1}-G_{t2}\right){⋃}^{}\left(G_{t3}-G_{t4}\right))$$

where the level parameters (*t*_1_ *t*_2_, *t*_3_ and *t*_4_) should be monotonically increasing or decreasing to confirm that generated sheets would not intersect with each other. The wall thickness of each sheet is dependent on the pair of level parameter, namely *t*_1_ and *t*_2_ as well as *t*_3_ and *t*_4_. It is well known that the biological structure of human tissues is inherently heterogeneous that arises from the hierarchical, multi-scale and grading structural distribution [41]. Here, the multi-sheet structure can be potentially used in the design of FGS to enhance the diversity and complexity.

**Figure 2:**
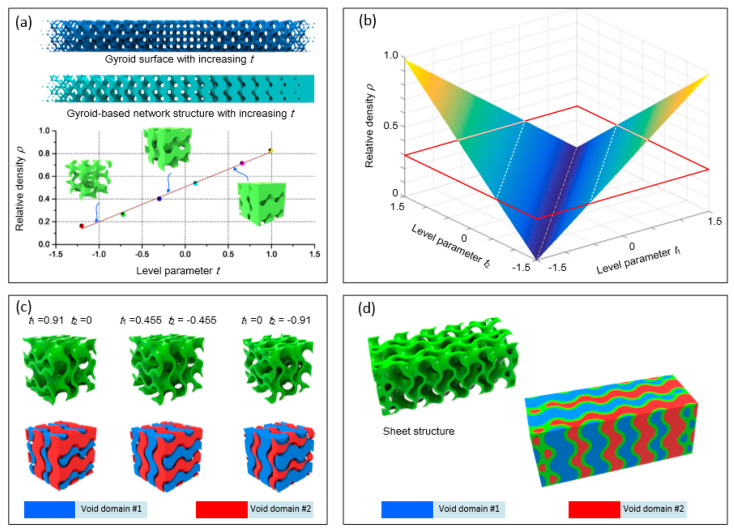
(**a**) Effects on the RD of level parameter in network-based structure; (**b**) relationship between RD and level parameters in sheet-based structure; (**c**) sheet structure with same RD constructed by different groups of level parameters; and (**d**) sheet structure with constant RD by grading level parameters.

The key for the parametric design of multi-sheet structure is to choose proper level parameters to achieve required properties, such as relative density (RD) and SA/V ratio. Therefore, it is necessary to investigate the effects of level parameters on these properties. A sheet structure can be considered as the combination of two TPMSs with the same cell size distribution and different level parameters (not necessarily the opposite numbers), thus it is reasonable to analyze from the network structure, which is constructed with only one TPMS. The relationship between RD and level parameter of Gyroid-based network scaffold has been investigated and is illustrated in Figure 2a. An approximately linear relation between them can be observed and a function is fitted as expressed in Equation (4).

(4) $$\rho_{network}=0.33\times t+0.5$$

Simply, the RD of a sheet structure with two TPMSs of level parameters *t*_1_ and *t*_2_ can be described as

(5) $$\rho_{sheet}=0.33\times \left|t_{1}-t_{2}\right|$$

Based on Equation (5), two symmetric surfaces which describe the RD of the generated sheet structures determined by two level parameters is shown in Figure 2b; it can be noticed that one sheet structure with a specified RD can be constructed with many pairs of level parameters. For example, if the expected density is 0.3 and a plane ρ = 0.3 is used to intersect with the surfaces, two symmetric lines would be obtained on the surfaces. Any pair of level parameters on the lines can generate a sheet structure with the density of 0.3. The volume of the voids on both sides of the generated sheet can be expressed as 1 − ρ*_t_*_1_ and ρ*_t_*_2_, respectively, with the assumption of *t*_1_ > *t*_2_, where ρ*_t_* is the RD of network-based structure with level parameter of *t*. Combined with Equation (4), they can be denoted by 0.5 − 0.33*t*_1_ and 0.33*t*_2_ + 0.5. Thus, the two generated voids are the same only if *t*_1_ = −*t*_2_.

Figure 2c shows three sheet structures with the same RD of 0.3, which requires the two level parameters should meet the following requirement: 0.33 × |*t*_1_ − *t*_2_| = 0.3. The level parameters of the first structure are 0.91 and 0, the volume ratio of the inside void (red domain) is 0.5 and the volume ratio of the outside void (blue domain) is 0.2. The two void volumes are the same in the second structure as the two level parameters are opposite numbers. The level parameters of the third sheet structure are opposite numbers to those of the first structure, so the inside volume ratio is 0.2, while the outside volume ratio is 0.5. Therefore, the sheet structure with a specified constant RD can be designed into diverse topologies by varying two level parameters simultaneously. A sheet structure with the constant RD of 0.3 is shown in Figure 2d; its level parameters change from (−1.21, −0.3) to (0.3, 1.21) along z-axis direction. It can be observed that the two void domains are changing gradually, resulting in a grading surface area and shape of two voids, which can be used for modulating the permeability of the two separate and self-interconnected pores.

**Figure 3:**
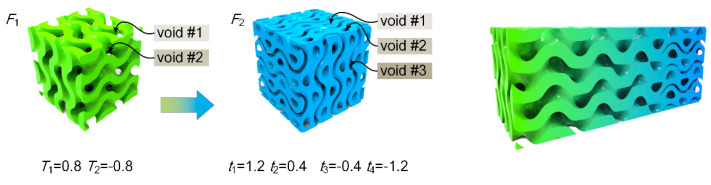
Generation of multi-sheet structure by transition between structures to generate branched properties.

With the control over the sheet topology, it becomes available to generate multi-sheet structures by appropriately designing the level parameters for each sheet. For example, two sheet structures with the level parameters of (−1.2, −0.4) and (0.4, 1.2), respectively, have approximately the same RD (0.264) and wall thickness; they can be integrated into a two-sheet structure as illustrated in Figure 3. There are three voids inside the generated structure; the volume ratios of the three voids are 0.104, 0.264 and 0.104 respectively. The mathematical expression of the two-sheet structure can be described by:

(6) $$F_{2}=\left\{\left(x,y,z\right)|\left(G<t_{1}\;\&\&\;G>t_{2}\right)||\left(G<t_{3}\;\&\&\;G>t_{4}\right)\right\}$$

where *t*_1_ and *t*_2_ are level parameters for one sheet and *t*_3_ and *t*_4_ are level parameters for another sheet. In this example, *t*_1_ = 1.2, *t*_2_ = 0.4, *t*_3_ = −0.4 and *t*_4_ = −1.2. Similarly, the single sheet structure can be simply expressed by:

(7) $$F_{1}=\left\{\left(x,y,z\right)|G<T_{1}\;\&\&\;G>T_{2}\right\}$$

where *T*_1_ and *T*_2_ are level parameters defining the sheet. To confirm a same RD with the two-sheet structure (0.528), *T*_1_ and *T*_2_ are set as 0.8 and −0.8 here.

More than one TPMS-based structures can be used to create a multi-morphology structure with smooth transition between one another [42]. The transition between structures (*F*_1_ and *F*_2_ here) can be easily achieved via the following mathematical operation:

(8) $$F=\varphi F_{1}+\left(1-\varphi \right)F_{2}$$

where *φ* serves as a weight function controlling the change in the transition region with a value between 0 and 1. Different weight functions would result in different topologies in the interface. In this study, a simple linear relationship was used, which can be described by the function:

(9) $$\varphi =\frac{\sqrt{{\left(x-x_{1}\right)}^{2}+{\left(y-y_{1}\right)}^{2}+{\left(z-z_{1}\right)}^{2}}}{\sqrt{{\left(x-x_{1}\right)}^{2}+{\left(y-y_{1}\right)}^{2}+{\left(z-z_{1}\right)}^{2}}+\sqrt{{\left(x-x_{2}\right)}^{2}+{\left(y-y_{2}\right)}^{2}+{\left(z-z_{2}\right)}^{2}}}$$

where (*x*_1_, *y*_1_, *z*_1_) and (*x*_2_, *y*_2_, *z*_2_) are two control points, between which the structure changes from *F*_1_ to *F*_2_. A hybrid structure transiting from one-sheet to two-sheet is shown in Figure 3. Based on the above elucidation, generation of transition between variable-sheet structures can be realized to achieve branched gradients.

### 2.2. Design of Case Studies

**Figure 4:**
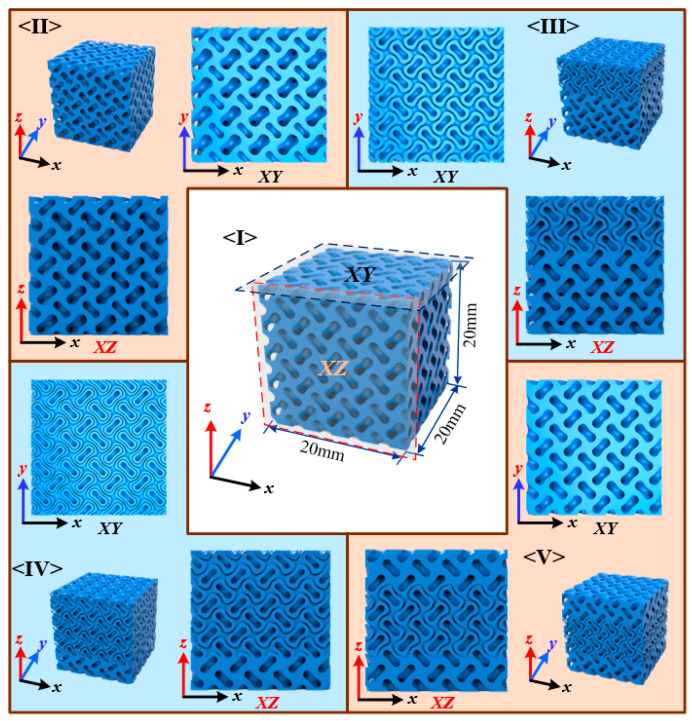
Five different types of sheet structure for experimental investigation.

Five different sheet structures with the same theoretical RD were designed and adopted to investigate the effect of sheet numbers on the obtained properties as shown in Figure 4. The first was a uniform sheet structure with a RD of 0.528, and the two level parameters were kept constant at 0.8 and −0.8. The second was another sheet structure with grading level parameters but constant wall thickness by confirming a constant gap between two level parameters. The level parameters were linearly grading from 1.1 and −0.5 to 0.5 and −1.1 to guarantee a constant average density of 0.528. In the third, a graded structure from single sheet to double sheets with the average density of 0.528 was designed. The level parameters of the one sheet structure was 0.8 and −0.8, while the two sheets structures were 1.1/0.3 and −0.3/−1.1. The two control points were located at 1/3 and 2/3 of the edge length along the grading direction (*z*-axis), and the weight function *φ* was calculated based on the distance along *z*-axis of each point to the control points. The last two structures were generated with different grading strategies. All specimens had the same average RD while different topologies. The edge length of the unit cell was 4 mm and each edge of the sample contained five unit cells.

### 2.3. Fabrication and Characterization

The designed models were triangulated using marching cube algorithm and then exported as STL files using *C#* codes, and *Materialise Magics* was used to repair the files. All specimens were 3D printed using a selective laser melting (SLM) machine (LeiJia, Guangzhou, China) with the material of 316L stainless steel, which is a widely used material in SLM. The elastic modulus of 316L is around 206 GPa, the yield strength is about 267.2 MPa and the density is 8.0 g/cm^3^. All fabricated samples were carefully cleaned through a sandblasting post-treatment to remove adhered powder particles. The additively fabricated samples were observed using a digital microscope (Axio imager A2m, Zeiss, Germany) to investigate their surface morphologies, including microscopic appearance and sheet thickness.

The fabricated specimens were subjected to static compression using a MTS testing machine equipped with a maximal load capacity of 200 KN, and the loading direction was in accordance to the building orientation. All samples were compressed at a loading rate of 2 mm/min to obtain the nominal stress–strain curve by recording the displacement and reaction force from each compression test. Five samples were tested for each design.

## 3. Results and Discussion

### 3.1. Property Evaluation Based on Designed Models

**Figure 5:**
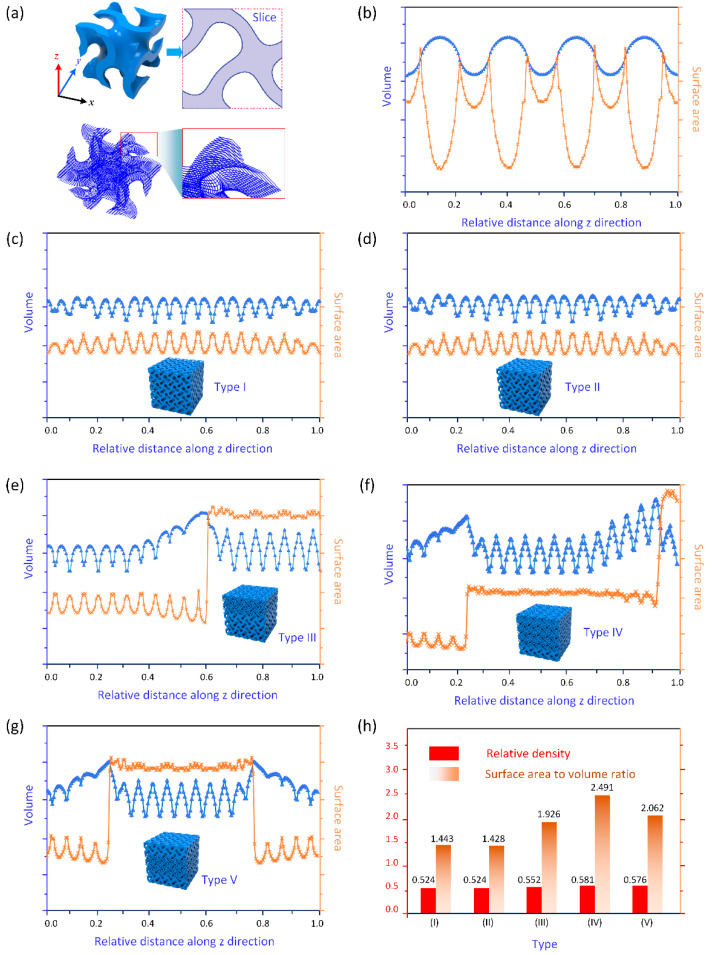
(**a**,**b**) Illustration of the slice-based method for evaluation of RD and surface area with a Gyroid sheet unit cell; (**c**–**g**) RD and surface area of Types I–V using slice-based method; and (**h**) RD and surface area based on digital models.

The RD and SA/V ratio are two essential parameters for porous structures. In this study, their variations along the grading direction were studied to evaluate the influence of the multi-sheet topology. A slice-based method was adopted here for approximately representing the RD and surface area. Specifically, the RD is the ratio of the solid volume to the scaffold volume, thus it can be obtained from all the cross-sectional surfaces by dividing the area of filled region by the total cross-sectional area. On the other hand, the change of surface area can be analyzed by calculating the perimeter of each slice. As illustrated in Figure 5a, a Gyroid sheet unit was sliced into several layers, the solid volume can be integrated by the filled area of all the slices and the surface area can be combined by the contour length of all the slices. It should be noted that the contours on the outer boundary should be excluded as they are not located on the surface of the scaffold. The variation of the solid volume and the surface area along *z* direction can be seen in Figure 5b. Periodic changes took place as expected for both variables due to the geometric characteristic of TPMS.

Subsequently, the solid volume and surface area of all the designed samples were obtained using the abovementioned slice-based approach and the relative values for each sample from the bottom to top were illustrated in Figure 5c–g, respectively. The uniform distribution of the Gyroid single-sheet unit resulted in steady solid volume and surface area as expected in Figure 5c. For Type II, despite the simultaneous change of two level parameters, the sheet thickness was confirmed with unchanged because of a constant difference between two level parameters. Hence, the variations of both solid volume and surface area were in accordance with that of Type I. The branched structure in Type III led to a significant increase of the surface area as shown in Figure 5e. The RD experienced a slight increase and then went down when the sheet structures transited from single sheet to two sheets. Thus, the proposed multi-sheet structure could be used to tune the surface area, while with a relatively constant RD to achieve desired biological and mechanical properties. Further, if the sheet numbers continuously increased to four, the surface area would rise again, as illustrated in Figure 5f. The last sample in Figure 5g shows that the RD and surface area could change accordingly with the change of the sheet numbers. At last, the RD and the SA/V ratio of each sample are provided in Figure 5h. It can be observed that their RDs were almost at the same value of 0.528 as designed. Slight deviations could be attributed to the transition between different structures, and the approximate representation of digital models would also bring errors. However, the SA/V ratio were significantly affected by the structures. Specifically, the SA/V ratio increased from 1.43 to 1.93 mm^2^/mm^3^ when the sheet structures changed from uniform single sheet to branched structures of Type III, and it continuously increased to 2.49 mm^2^/mm^3^ when the sheet numbers changed from one to four within a scaffold. These results reveal that the proposed method could effectively generate structures with grading SA/V ratio by controlling the sheet topologies.

### 3.2. Physical Characterization of Fabricated Specimens

**Figure 6:**
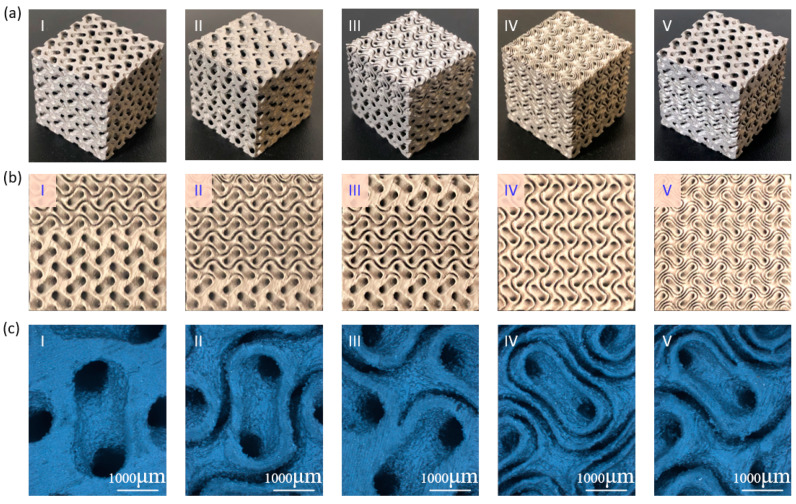
Microstructural characterization of manufactured specimens with different designs: (**a**) fabricated specimens; (**b**) side/top views of the fabricated parts; and (**c**) close-up views of the surfaces.

All additively manufactured specimens, as shown in Figure 6a, were weighted using a digital balance and their actual RDs were calculated based on the average measured mass for each design and the bulk density of 7.9 g/cm^3^. The actual RDs were compared with theoretical values based on the models, as listed in Table 1. The difference in RD between design and model was attributed to the modeling process mentioned in Section 3.1, while the deviation between theoretical and actual RDs should be attributed to the manufacturing process. Different process-related factors, including contour scanning and powder melting, would cause errors [6]. Errors of all specimens were smaller than 2%, indicating that the designed sheet structures could be fabricated with desirable accuracy using SLM process.

The change in sheet topologies can be seen in Figure 6b by capturing the surface of manufactured structures. In Figure 6b, I–III show the front surface of Samples III–V, respectively, and the corresponding variations in the thickness and pore size could be observed. All structures had smooth transition regions between different sheet numbers with three different grading methods. The top surface of Samples III and IV is also provided in Figure 6b (IV–V) to indicate that multi-sheet structure could be obtained with high resolution. Both the sheet thickness and pore size were varied when the sheet number changed. For example, the sheet thickness was about 600 μm when the sheet number was one, while the thickness decreased to about 300 μm when the sheet number increase to two and continuously reduced to around 150 μm when the sheet number was four. With the design of level parameters, the average RD of each specimens was almost unchanged with the changing sheet numbers. As such, the change in the pore size due to the grading sheet numbers could be deduced. At the same time, the gap between sheet structures was also varied, severing as a potential variable to control the geometric morphologies of the multi-sheet structures. Close-up view images are shown in Figure 6c to further demonstrate the smooth transition between different sheet structures.

### 3.3. Mechanical Performance Under Compression

**Figure 7:**
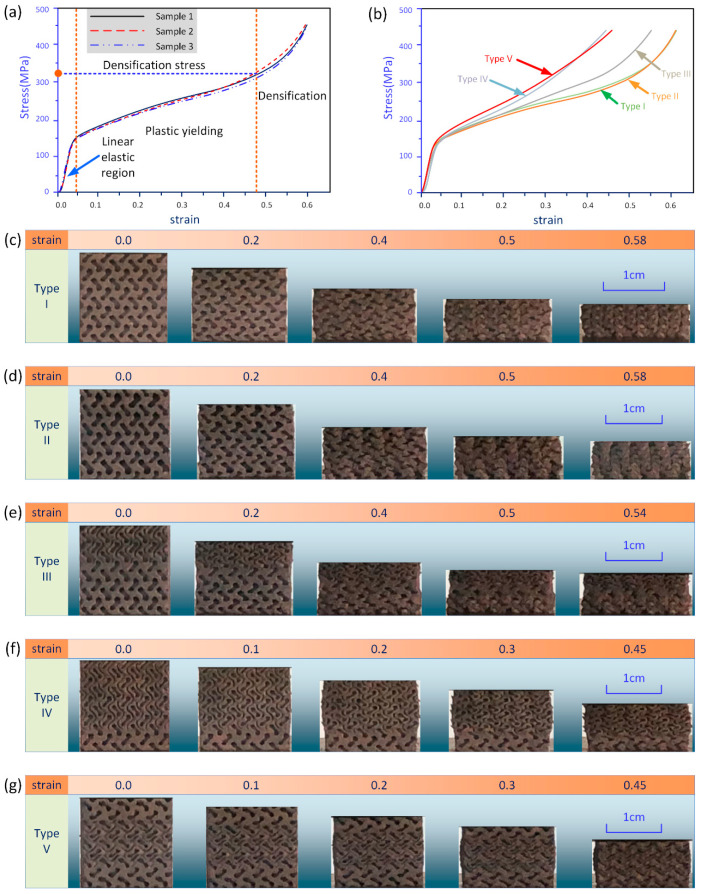
Stress–strain response and compressive behavior of specimens with different design: (**a**) illustration of the obtained stress–strain curve of one design; (**b**) obtained stress–strain curves of five different designs; and (**c**–**g**) compression deformation of different types of specimens.

All specimens were compressed with a loading direction parallel to the manufacturing direction to eliminate the impact on the mechanical performance caused by the layer-based fabricating process. The compression process was terminated when the strain reached to 0.6 or the loading force was beyond a preset limit (180 KN in the experiment). The corresponding compressive stress–strain responses of each design are shown in Figure 7b. The stress was calculated from the load on the nominal surface area of the structures (2 cm × 2 cm here). Actually, it should be noted that the stress and strain distribution varied within the specimens along the loading direction due to the grading properties and the inconsistent boundary edges at different heights.

Figure 7a illustrates the stress–strain curves of samples with ungraded design as a baseline. High agreement between three samples with the same design could be observed due to outperformed structural connectivity and consistency of the adopted AM techniques. In the stress–strain curves in Figure 7a, three different deformation stages can be identified. The compressive response began with a linear elastic stage, from which the elastic modulus could be determined based on the slope of linear portions. The first stage was mainly governed by the bending of structural properties [43]. After that, the stress–strain curves started to enter a plateau region at around 0.05 strain resulting from the progressive collapse of the porous materials. At last, another rapid increase of the stress occurred due to the contact and squeeze of materials against each other for densification.

The stress at the point where the curve turned from plastic yielding stage to densification stage was termed as densification stress, which could be used to evaluate the energy absorption capability [44]. Figure 7b shows that the densification stress for samples of different designs was distinct. Specifically, the average densification stress for specimens of one sheet, either with uniform level parameter (Type I) or grading level parameter (Type II), were both found to be about 320 MPa, implying that the impact on the compressive properties of grading level parameters was so minimal that could be neglected. At the same time, their stress curves had a smoother transition into material densification because the pore size and sheet thickness were almost unchanged along the loading direction, and their RDs were the smallest in all sample designs. As for specimens with simple gradient (Type III), a larger densification stress can be found due to the multi-sheet structure at the top region. The smaller pore size and closer gap between sheets would enhance the hardening behavior and its RD was slightly larger than that in the first two designs. As such, the fabricated samples with other two different gradients (Types IV and V) had a much larger densification stress due to the highest RD. The difference between the last two designs was the sheet structures at the top domains, but their compressive response was similar. Therefore, the mechanical properties were mainly determined by the average RD of the structures. Moreover, some other associated mechanical properties, including elastic modulus and yield stress, were close between different designs as they had close RDs.

Figure 7c–g illustrates the deformation stages of specimens with five different designs from their original shapes up to maximal strains. Different deformation behaviors resulted in different shapes of the compressed samples. The overall shapes of ungraded sheet structures (Types I and II) were almost unchanged with the increasing width due to the Poisson effect. However, a trapezoidal shape was found in the Type III because of the grading distribution of the sheet numbers. Furthermore, barrel shaped edges could be found in the last two samples as the Poisson’s ratio between layers with different sheet morphologies were different.

### 3.4. Design of Functional Gradients with Multi-Sheet Structures

Based on the above findings of the proposed branched sheet structures, it becomes available to harness the potential of functional gradients for specific applications. The main contribution of the branched structure is to tune the SA/V ratio significantly without changing the average RD, which imposes a larger impact on the mechanical properties. Therefore, FGS with particular distribution of mechanical constraints and physical properties can be designed by combining several parameters in the design process. For example, the RD of a sheet structure can be effectively controlled by the level parameters of unit cells, and thus the desired mechanical performance can be achieved. The effects on the mechanical properties of RD have been widely investigated [30,45], thus it is simple to obtain the mapping relationship between the design parameters and the mechanical properties. Three different functional graded structures were explored by simultaneously considering RD and SA/V ratio.

**Figure 8:**
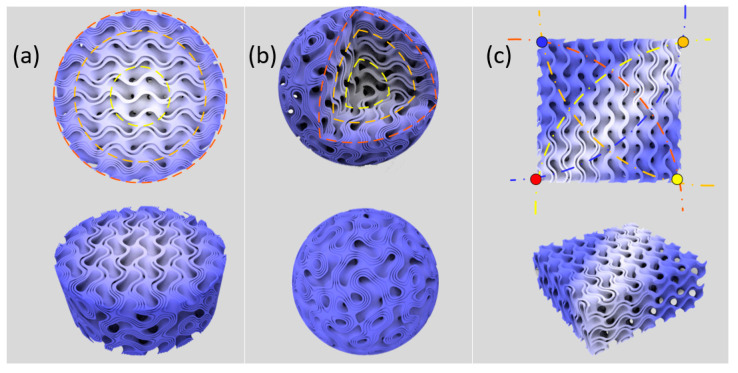
Designs of 2D and 3D functional gradients using the multi-sheet structure: (**a**) 2D gradient of a cylinder; (**b**) 3D gradient of a sphere; and (**c**) 2D heterogeneous design.

The first example was a simple 2D gradient, as illustrated in Figure 8a. A circle was partitioned into several rings based on the distance to the center. The RD of each ring was specified to obtain a grading distribution to match the exact properties and construct a biomimetic functionally gradient. The RDs of three rings were 0.264, 0.33 and 0.396, respectively, in the example. Meanwhile, the sheet numbers for each ring were determined based on the requirement on the SA/V ratio. Then the level parameters for each sheet could be obtained based on the specified RD and sheet numbers. The substructures in each region would be accordingly generated and smoothly transitioned in between using the linear weight function. The second example illustrated in Figure 8b was a sphere, which was divided into several layers, whose design parameters were all specified and same with the first example. The last example was designed based on a 2D heterogeneity. Design parameters, including RD and sheet numbers at four corners of a square, were specified in advance. Here, the coordinates of corners were the control points in the design process. The key was to combine these four substructures with smooth transitions while the properties at each corner could be preserved. To achieve this, a sigmoid function method [26] was adopted to calculate the relative weight of four substructures according to the distance to four control points. Thus, any number of substructures could be combined into whole functional graded structures using this method. These generated graded structures showed the potential of the proposed TPMS-based branched features in constructing complex and bionic structures.

## 4. Conclusions

In this study, TPMS-based sheet structures were adapted into branched features to construct hierarchical porous bone scaffolds. The difference between two level parameters of a sheet determines its RD, but its morphology can be varied by simultaneously changing the two level parameters. Consequently, more than one sheet structures with the same distribution of unit cell size but different level parameters can be integrated into a multi-sheet structure without mutual or self-intersections. Further, different multi-sheet structures can be used to generate multi-morphology materials using linear weight functions controlling the transition between different structures. Different branched sheet structures investigated in this paper were parametrically designed via this method. The RD and SV/A ratio of samples were studied and compared using a slice-based approach to verify the feasibility and versatility of the branched sheet structures. After additively fabricated by SLM, the geometric morphology of all the samples were observed to confirm the manufacturability. Moreover, compression tests were conducted to evaluate the mechanical properties from the obtained strain–stress curves, and the deformation behavior was also analyzed form the captured images at different strain levels. Overall, the feasibility of the proposed branched sheet structure was demonstrated and verified.

Clearly, there is a great potential for the proposed TPMS-based branched structures to provide more versatile properties, such as tunable permeability and mechanical properties. Furthermore, the proposed structures can be easily extended to other AM techniques with other biomaterials, such as extrusion-based technique [46] and projection stereolithography [47] for more advanced TE applications.

However, more detailed work is required to comprehensively evaluate the proposed grading structures and thus demonstrate its full potential. For example, effects on cellular infiltration and permeability of the sheet morphologies should be experimentally investigated. More importantly, both in vitro and in vivo studies should be further conducted to evaluate the actual biological performance in bone TE.

## Figures and Tables

**Table 1:** Analysis of RD for fabricated specimens.

Type	Design RD	Model RD	Average Mass (g)	Actual RD	Error
I	0.528	0.524	32.65	0.517	−1.3%
II	0.528	0.524	32.56	0.515	−1.7%
III	0.528	0.552	34.62	0.548	−0.7%
IV	0.528	0.581	36.82	0.583	0.3%
V	0.528	0.576	36.29	0.574	0.3%
